# Counseling on family planning during ANC service increases the likelihood of postpartum family planning use in Bahir Dar City Administration, Northwest Ethiopia: a prospective follow up study

**DOI:** 10.1186/s40834-018-0081-x

**Published:** 2018-12-27

**Authors:** Tadese Ejigu Tafere, Mesganaw Fanthahun Afework, Alemayehu Worku Yalew

**Affiliations:** 1 School of Public Health (SPH), College of Health Sciences, Addis Ababa University, Addis Ababa, Ethiopia; 2 School of Public Health, College of Medicine and Health Sciences, Bahir Dar University, Bahir Dar, Ethiopia; 3 School of Public Health, College of Health Sciences, Addis Ababa University, Addis Ababa, Ethiopia

**Keywords:** Postpartum family planning use, Unmet need for family planning, Postpartum period

## Abstract

**Background:**

Closely spaced pregnancies within the first year postpartum increases the risk of death for both the mother and baby. Many countries recommend providing pregnant women with post-partum family planning counselling during antenatal care visits. However, data on the extent to which providers utilize these opportunities and the role of family planning counseling during antenatal care in promoting the use of postpartum modern family planning remain limited especially in developing countries. Therefore, this study was aimed at investigating the role of family planning counseling during antenatal care in promoting postpartum modern family planning use within 6 weeks after birth.

**Methods:**

Nine hundred seventy pregnant women with gestational age ≤ 16 weeks who came for their first Antenatal Care (ANC) visit were enrolled and followed until 6 weeks after delivery. Longitudinal data was collected during consultation with ANC providers using structured observation checklist to assess whether or not the providers counsel pregnant women on post-partum family planning use during their four focused ANC visits. Exit interview was also conducted at 6 weeks after they gave birth when they came to immunize their child to assess whether they were starting to use postpartum modern family planning. Completed data were obtained from 823 women. Generalized Estimating Equation was carried out to identify predictors of postpartum modern family planning use by controlling the cluster effect among women who received ANC services in the same health facility.

**Results:**

Postpartum modern family planning use within 6 weeks after delivery among the study women was 157(19.1%) with 95%CI (16.4, 21.9); Among 187 pregnant women who were counseled at least once, 72(38.5%) of them used post-partum modern family planning compared to 13.4% of post-partum women who were not counseled at all (*p* < 0.001). Counseling about postpartum family planning during antenatal care, satisfaction on the antenatal care services women received while they were pregnant, counseling on birth preparedness and complication readiness plan, counseling on breast feeding and post-natal care use were independent predictors for postpartum modern family planning use.

**Conclusion:**

Less than one in five post-partum women were using postpartum family planning within 6 weeks after birth. Family planning counseling during ANC services had a significant effect on promoting postpartum modern family planning use. Therefore, health providers need to ensure continuity of care through strengthening integration of family planning counseling services during ANC and referral linkages between community and health workers.

## Plain English summary

Immediate post-partum family planning use helps women to space their pregnancies, lowering their risk for low birth weight and premature birth, both linked with infant mortality. In Ethiopia, nearly half (47%) of all pregnancies occurred within < 24 months after preceding birth; To what extent providers utilize opportunities of ANC visits for family planning counseling to promote the use of postpartum modern family planning remain limited especially in developing countries.

Pregnant women with gestational age ≤ 16 weeks who came for their first ANC visit were enrolled and followed until 6 weeks after delivery. Longitudinal data was collected during consultation with ANC providers to assess whether or not the providers counsel pregnant women on postpartum family planning use during their four focused ANC visits. Exit interview was also conducted at 6 weeks after they gave birth when they came to immunize their child to assess whether they were starting to use postpartum modern family planning.

Among 823 pregnant women, 187(22.7%) were counseled about post-partum family planning at least once during their four ANC visits and postpartum modern family planning use within 6 weeks after delivery was 157(19.1%). Injectable and implants were the most common adopted modern post-partum family planning methods.

A fraction of pregnant women was provided with family planning counselling during antenatal care that led to lower level of adoption of post-partum family planning within six weeks after delivery which implied a need to ensure integrity of maternal health services during antenatal care.

## Background

Family planning counselling during the antepartum period provides a crucial window of opportunity to address unmet need for family planning during the post-partum period. Rates of maternal and infant mortality and morbidity could be substantially decreased if unplanned and unwanted pregnancies were prevented and if all new pregnancies could be spaced at least two years after a birth [1].

Family planning can avert more than 30% of maternal deaths and 10% of child mortality if couples space their pregnancies more than two years apart [2, 3]. On the contrary, closely spaced pregnancies within the first year postpartum increase the risk of death for both the mother and baby, resulting in increased risks for adverse outcomes, such as preterm birth, low birth weight and small for gestational age [4].

The period after delivery is complex and challenging, during which a woman has to care for her newborn child as well as cope with psychological and physical changes. Focused antenatal family planning counseling increased the possibility postpartum family planning use [5].

The service delivery guidelines for antenatal care services in many countries recommend providing pregnant women with counseling concerning post-partum family planning, breastfeeding, danger signs birth preparedness and complication readiness plan and other topics. However, only a fraction of clients in Sub-Saharan Africa(SSA) receive information on any of these topics during their antenatal care visits [6].

In five countries of Latin America and the Caribbean, about half of pregnant women receive some family planning information during antenatal care visits [7]. In Sub-Saharan Africa, the proportion of postpartum women who are exposed to the risk of pregnancy by having sex while using no family planning method within two years after childbirth was nearly one third [8, 9].

In Ethiopia, evidence has been found that nearly half (47%) of all pregnancies occurred within a short birth interval of less than 24 months after the preceding birth and 86% of post-partum women within six months have unmet need for family planning [10].

Postpartum women are an important target groups because they may not realize that they are at risk of pregnancy even if they are breastfeeding [11]. However, 37% of sexually active women are at risk of pregnancy during the first six months postpartum, this risk increases to 64% among women 6–11 months postpartum [10].Therefore, concentrating efforts on increasing postpartum family planning use among these women could have a proportionally greater impact than focusing attention on other populations.

A very limited number of studies examined the role of ANC as a facilitator for post-partum modern family planning use (short/long acting hormonal contraceptives, permanent family planning techniques or barrier methods like male or female condom use). Those studies were cross sectional focused on the assessment of the relationship between the frequency of ANC visits rather than the antenatal care family planning counseling and post-partum family planning use. In addition those who assessed the association between provision of antenatal care family planning counseling during any of their ANC visits and postpartum family planning use were based on the women’s’ self-report and they also assessed post-partum family planning use within one or two years after birth rather than within the six weeks postpartum which is the WHO recommendation [12, 13].

Therefore, this follow up study was intended to bring an insight about the role of family planning counseling during the four focused antenatal care visits in promoting the use of modern family planning use within 6 weeks after birth. More over to explore the relationship between other contents of antenatal care services (like counselling on birth preparedness and complication readiness plan, counselling on breast feeding, satisfaction on ANC) and the use of post-natal care with post-partum modern family planning use. The findings of this study will provide credible evidence for maternal and child health program implementers and policy makers to reduce maternal and child mortality.

## Methods

### Study design, settings and population

A facility based prospective follow up study was conducted from October 2015 to August 2016 in seven public health facilities; one hospital and six health centers of Bahir Dar City Administration, Amhara National Regional State, which is located in the North West part of Ethiopia. According to the Amhara National Regional State Bureau of Finance and Economic Development report, the projected population by 2015/16 was 297,775 (80.5% urban Vs 19.5% rural), of these 156,515 (52.6%) were females and there were 10,035 eligible pregnant women in the same year [14]. All first visit pregnant women with gestational age ≤ 16 weeks attending antenatal care services during data collection period who were selected by systematic random sampling (k = 3) and voluntary to participate in the study were included. Pregnant women were followed from their first to fourth antenatal care visits while they received their antenatal care services to investigate whether health professionals provide them with postpartum family planning counseling. Exit interview was also conducted just after their fourth ANC visit to measure their satisfaction on the ANC service they received and at 6 weeks after birth when they came to immunize their child to assess whether or not they started to use postpartum modern family planning and to identify the type of postpartum modern family planning. The satisfaction scores were included to provide insight on whether satisfactory scores influenced postpartum family planning use. The detail of the method is described elsewhere.

### Sample size and sampling technique

The current study is part of a follow up study with multiple objectives. The detail of the sample size calculation to address all objectives (considering the maximum adequate sample size which was 970) and the sampling procedure is described elsewhere [15]. For the current study a sample size was calculated with 2.8% postpartum modern family planning use among women who had no ANC (considering these women as they were not counseled on postpartum family planning during ANC) [16] and 5% difference in post-partum modern family planning use among women who were counseled, 80% power, 5% level of significance and 10% non-response rate. The final calculated sample size was 777. However, to increase the power of the study, all 823 women who completed the follow up period were included in the analysis.

### Data collection process

Seven female diploma midwives and two female Bachelor of Science midwives who were not the staffs in the study health facilities were recruited as data collectors and supervisors respectively to observe whether or not the ANC provider counsels a woman about postpartum family planning during each of the four ANC visits and provide other contents of ANC services.

To measure the satisfaction of pregnant women on ANC services they received during their pregnancy period an exit interview was conducted just after their fourth ANC visit using eight items. Each item has five point Likert scales from strongly disagree (1) to strongly agree (5).

In addition, there was also another exit interview with women at 6 weeks after birth when they came to the health facility to immunize their child to assess whether or not they were using a modern family planning. If a woman doesn’t come to the health facility for immunization, the data collectors were expected to trace her at home based on her address registered during the first visit.

#### Dependent

Post-partum modern family planning use (yes/no).

#### Independent

Counseling on postpartum modern family planning during each of the four ANC visits was the main exposure variable and the other independent variables include: satisfaction on the ANC services that women received while they were pregnant, counseling services given about birth preparedness and complication readiness plan, breast feeding and immunization; postnatal care use and place of delivery.

The satisfaction score of pregnant women on the ANC services they received was computed from the eight items aimed to assess privacy, confidentiality, costs related to ANC service, client-provider interaction, their perception about ANC services provided and whether they recommend the health facility for others or not. The items had internal reliability (Cronbach’s α of 0.74). A respondent had a minimum of 8 and a maximum of 40 points on ANC satisfaction score. The satisfaction score was converted to percentage satisfaction score to make it standardized [17]. The percentage mean satisfaction score for client satisfaction on the ANC services received was 73.2. Clients were categorized as satisfied (if they score above the percentage mean satisfaction score) otherwise not satisfied.

### Data analysis

Data were coded and entered into EPI data version3.1 and exported to SPSS version 20 for analysis. Descriptive statistics were used to describe the data. Generalized Estimating Equation logistic regression analysis with robust estimator and exchangeable working correlation matrix was carried out to control the cluster effect of women who received ANC services within the same health facility and to identify the predictor variables for postpartum modern family planning use at 6 weeks after birth. A *p*-value < 0.2 was considered to retain variables for the multivariable Generalized Estimating Equation logistic regression analysis. And *P*-value < 0.05 was considered to identify statistically significant predictors for postpartum modern family planning use.

## Results

### Characteristics of study participants

Among 970 pregnant women who were enrolled to the larger study 823(84.8%) completed the follow up period. The reasons for 15.2% loss to follow up were abortion, self-referral to other health facilities permanent change in work place and the long distance to reach health facilities especially when the gestational age increases. There was no statistically significant difference in family planning counseling service during ANC between those women who completed the follow up and those who were loss to follow up. The mean age (±standard deviation) of 823 women who completed the follow up period was 25.46 ± 4.37 years. Nearly 90 % of women were urban residents (88.5%) and Orthodox Christian (90.9%). With regard to their educational status, 21.0% of women had no formal education while 57.5% of women had completed secondary school and above (Table 1).

**Table 1:** Socio-demographic characteristics and postpartum family planning counseling (PPFP) of ANC attendants at public health facilities of Bahir Dar City administration (*N* = 823), October 2015 to August 2016

Variables	PPFP counseling at least once during ANC		Total frequency
	Yes (%)	No (%)	
Age			
15–24 years	81(22.9)	273 (77.1)	354 (100.0)
25-34 years	101 (23.5)	328 (76.5)	429 (100.0)
≥ 35 years	5 (12.5)	35 (87.5	40 (100.
Residence			
Urban	174 (23.9)	554 (76.1)	728 (100.0)
Rural	13 (13.7)	82 (86.3)	95 (100.0)
Educational status			
No formal education	36 (20.8)	137 (79.2)	173 (100.0)
Primary	37 (20.7)	140 (79.1)	177 (100.0)
Secondary and above	114 (24.1)	359 (75.9)	473 (100.0)
Occupation			
Farmer	11 (12.4)	78 (87.6)	89 (100.0)
House wife	108 (25.5)	315 (74.5)	423 (100.0)
Employee^a^	68 (21.9)	243 (78.1)	311 (100.0)
Religion			
Orthodox	175 (23.4)	573 (76.6)	748 (100.0)
Muslim	12 (16.4)	61 (83.6)	73 (100.0)
Protestant	0 (0.0)	2 (100.0)	2 (100.0)
Marital status			
Married	184 (22.7)	627 (77.3)	811 (100.0)
Divorced	2 (20.0)	8 (80.0)	10 (100.0)
Widowed	1 (5.0)	1 (50.0)	2 (100.0)
Ethnicity			
Amhara	179 (22.7)	609 (77.3)	788 (100.0)
Tigre	6 (24.0)	19 (26.0)	25 (100.0)
Awi	2 (28.6)	5 (71.4)	7 (100.0)
Oromo	0 (0.0)	3 (100.0)	3 (100.0)
Grand total	187 (22.7)	636 (77.3)	823 (100.0)

^a^governmental employees, nongovernmental employees,daily laborers and waiters

### Family planning counselling during antenatal care and post-partum modern family planning use

Among 823 pregnant women, 187(22.7%) of pregnant women were counseled about postpartum family planning at least once during their four ANC visits. All pregnant women were not counseled about postpartum family planning during their first and second ANC visits; however, 60(7.3%) and 186(22.6%) of pregnant women were counseled about postpartum modern family planning during their third and fourth visits respectively.

The magnitude of postpartum modern family planning use within 6 weeks after delivery among the study women was 157 (19.1%) with 95%CI (16.4, 21.9) though the contraceptive methods like IUCD were provided in the immediate post-partum period before discharge in the same room. Injectable, Implanon, pills and IUCD were used by 111(13.5%), 34 (4.1%), 10(1.2%) and 2(0.2%) post-partum women respectively.

Among 187 pregnant women who were counseled at least once during their ANC visits, 72(38.5%) of them used postpartum modern family planning compared to 13.4% of postpartum women who were not counseled at all during their four ANC visits (*p* < 0.001).

With regard to satisfaction on the ANC service provision, among 376(45.7%) women who were satisfied with their ANC service, 111(29.5%) were using postpartum family planning. However, among 447(54.3%) who were not satisfied by the service, only 46(10.3%) were using postpartum modern family planning (*p* < 0.001) in their postpartum period within 6 weeks after delivery.

### Factors associated with postpartum modern family planning use

In a Generalized Estimating Equation binary logistic regression analysis, there was a positive association between postpartum modern family planning use and family planning counselling during ANC, satisfaction on the ANC service, birth preparedness and complication readiness plan, place of delivery, post-natal care service utilization, counselling on breast feeding, residence, educational status and occupation of the mother.

In multivariable Generalized Estimating Equation logistic regression analysis, since post-natal care use and place of delivery showed co linearity effect, place of delivery was not fitted in the final model. Family planning counselling during antenatal care, birth preparedness and complication readiness plan, counselling on breast feeding at least once during ANC visits, satisfaction on the antenatal care service while she was pregnant and post-natal care visit remained to have statistically significant association with postpartum modern family planning use.

The odds of postpartum modern family planning use within 6 weeks after birth among women who were counseled about postpartum family planning at their third or fourth visit were 3.5 times higher compared to those who were not counselled at any of their visits (AOR = 3.5; 95% CI:2.19,5.49). The odds of postpartum modern family planning use within 6 weeks after birth among women who were counseled about birth preparedness and complication readiness plan at their third or fourth ANC visit were 2.2 times higher compared to those who were not counselled at all (AOR = 2.2; 95% CI:1.32,3.55). The odds of postpartum modern family planning use within 6 weeks after birth among women who were counseled about breast feeding at least once during their ANC visits were 1.8 times higher compared to those women who were not counselled during their ANC visits (AOR = 1.8; 95% CI:1.15,2.82). The odds of postpartum modern family planning use within 6 weeks after birth among women who had at least one post-natal care visit during their postpartum period were 13.5 times higher compared to those who had no any post-natal care visit ((AOR = 13.5; 95% CI:8.24,22.07). In addition, the odds of partum modern family planning use within 6 weeks after birth among women who were satisfied by the ANC service they received for their recent pregnancy were 4.1 times higher compared to their counter parts (AOR = 4.12; 95% CI:2.55,6.66) (Table 2).

**Table 2:** Multivariable GEE logistic regression results to assess the association between counseling on postpartum family planning (PPFP) during ANC and PPFP use at public health facilities of Bahir- Dar City Administration (*N* = 823), October 2015 to August 2016

Variables	Postpartum modern family planning use		COR(95%CI)	AOR(95%CI)
	Yes (%)	No (%)		
PPFP counselling during ANC				
Counselled at least in one visit	72 (38.5)	115 (61.5)	4.06 (2.80,5.89)	3.47 (2.19,5.49)*
Not counselled at all	85 (13.4)	551 (86.6)	1.00	1.00
Residence				
Urban	154 (21.2)	574 (78.8)	8.23 (2.57,26.34)	1,02 (0.33,3.12)
Rural	3 (3.2)	92 (96.8)	1.00	1.00
Educational status				
Secondary school and above	118 (24.9)	355 (75.1)	3.78 (2.10,6.77)	1.47 (0,67,3.25)
Primary school	25 (14.1)	152 (85.9)	1.87 (0.936,3.73)	1.12 (0.47,2.71)
No formal education	14 (8.1)	159 (91.9)	1.00	1.00
Occupation				
Employee ^a^	84 (27.0)	227 (73.0)	10.61 (3.27,34.46)	1.93 (0.50,7.48)
House wife	70 (16.5)	353 (83.5)	5.67 (1.75,18.49)	1.47 (0.41,5.32)
Farmer	3 (3.4)	86 (96.6)	1.00	1.00
Counselling on BPRCP				
Counselled at least in one visit	116 (26.9)	315 (73.1)	3.15 (2.14,4.64)	2.16 (1.32,3.55)*
Not counselled at all	41 (10.5)	351 (89.5)	1.00	1.00
Counselling on breast feeding				
Counselled at least in one visit	72 (32.6)	149 (67.4)	2.94 (2.04,4.23)	1.80 (1.15,2.82)*
Not counselled at all	85 (14.1)	517 (85.9)	1.00	1.00
At least one PNC visit				
yes	96 (60.4)	63 (39.6)	15.06 (9.97,22.80)	13.49 (8.24,22.07)*
no	61 (9.2)	603 (90.8)	1.00	1.00
Satisfaction on ANC service during pregnancy				
Satisfied	111 (29.5)	265 (70.5)	3.65 (2.50,5.32)	4.12 (2.55,6.66)*
Not satisfied	46 (10.3)	401 (89.7)	1.00	1.00

*Indicates significant difference at *p* < 0.05

^a^Government, private and Nongovernmental organization employees

## Discussion

Based on the 2016 Ethiopian Demographic and Health Survey (EDHS) report, with only 35% modern family planning use, and high unmet need (22%) [18], investigating the effectiveness of providing postpartum family planning information through antenatal care services on postpartum family planning use, and the extent to which providers utilize these opportunities, are increasingly important. For women who are not breastfeeding, pregnancy can occur within 45 days of giving birth [19].

The current study found that less than one out of five postpartum women were using modern family planning at 6 weeks of after birth. The finding is consistent with a study done in Adigrat Northern Ethiopia 24.6% [20] but is higher than a study done including different countries using their respective most recent (2008–2012 DHS data); Ethiopia (8%), Comoros(6.4%) and Burundi (5.8%) [21].

The possible explanations for higher postpartum family planning use compared to studies done in some of the afore mentioned countries could be attributed to the time gap between the current study and Demographic Health Survey (DHS) data collected in different countries; all participants of the current study had four ANC visits that might increase their tendency to use postpartum family planning and the difference in background characteristics of study participants, for instance most (88.5%) of the participants of this study were urban dwellers so that they might have better awareness and access to family planning methods compared to DHS studies that includes both rural and urban population groups. In addition, efforts by the Ethiopian government to strengthen strategies for maternal and child health mortality reduction like the different community based reproductive health services and health education being given on house to house basis by health extension workers might also have an effect for the increase in postpartum modern family planning use.

Among postpartum women who adopted postpartum modern family planning, Injectable and implants were the most common methods reported by the current study and this is also in line with the analysis done to assess method mix among PPFP users from DHS 2011 data [22] except that there is improvement in utilization of Implanon compared to EDHS 2011 report (21.7% Vs 10%). Similarly, studies done on the general population in Gondar and 2016 EDHS report also reported that the most commonly used PPFP methods are injectable and implants [16, 18] .

This implied method mix in Ethiopia relies heavily on short-acting methods, with the majority of women using injectable and only a fraction of postpartum family planning users using long-acting methods (implants and IUDs).

This study also revealed that even if less than one–fourth of the women were given counseling on postpartum family planning; information on family planning during antenatal care had significant effect on improving the use of postpartum modern family planning. This finding is also supported by studies done in Mexico [23], Uttar Pradesh [24] and systematic review published in 2014 that reported integration of family planning into antenatal care services showed increased modern family planning use where the demand is high [25].

This might be due to the reason that frequent exposure of pregnant women to health professionals during antenatal care offers ample opportunity to discuss about family planning when couples are not distracted by a new baby and when they have time to consider all the options.

Satisfaction by the ANC services during pregnancy was significant in predicting the likelihood of using postpartum modern family planning within 6 weeks by the current study. The study in Mexico among low-income women also supported this finding as it reported women living in communities with high quality care were more likely to use a family planning method than those in communities with lower quality of care [23].

This might be attributed to high quality care increases the confidence of women on the health system there by comply with the advice given by health professionals and might have a positive effect to use the continuum of care (antenatal care to post-natal care including postpartum family planning use).

The use of modern family planning is significantly associated with the use of post-natal care in this study. This relationship is also evidenced by other studies findings conducted in North West Ethiopia [16],India [26] and Mexico [23].

The present study showed women who were counseled about birth preparedness and complication readiness plan during antenatal care were more likely to use post-partum modern family planning compared to their counter parts. This might be due to the reason that when health professionals discuss about birth preparedness and complication readiness plan they are more likely to discuss with the danger signs of pregnancy and the probability of their occurrence in closely spaced pregnancy; which might have an influence on the decision of women to use postpartum family planning. Similarly, this study also reported women who were counseled about breast feeding were more likely to use postpartum modern family planning.

As the data collection was done by direct observation while women received antenatal care services using observation checklist adapted from WHO Basic Emergency Obstetric and Newborn Care (BEmONC) guideline, the study might not be affected by self-report bias.

However, ANC providers might maximize their effect due to the presence of data collectors (hawthorn effect) though its effect will decrease as the providers familiarize their presence since the data collection period was long (1 year). Since the focus of this study investigating the presence or absence of family planning counseling during ANC rather than about the standard/ quality of the counseling session, it might also have a limitation.

In addition, this study might also have selection bias as it was a facility based study and women who came to health facilities might have different characteristics from those who didn’t come.

## Conclusion and recommendation

A fraction (less than one-fourth) of pregnant women was provided with family planning counselling during antenatal care. Family planning counseling during antenatal care services had a significant effect on postpartum modern family planning use. Counseling about postpartum family planning during antenatal care, satisfaction on the antenatal care services women received while they were pregnant, counseling on birth preparedness and complication readiness plan, counseling on breast feeding and post-natal care use were independent predictors for postpartum modern family planning use.

Therefore, programme managers and health providers need to ensure continuity of care through coordination within the health system such as integrating family planning counseling services during antenatal care and providing referral linkages between community and health facility for antenatal care, postnatal care and postpartum modern family planning use to reach postpartum women when their demand for family planning is high.

## Abbreviations

ANC: Antenatal Care
AOR: Adjusted Odds Ratio
BPRCP: Birth preparedness and Complication Readiness Plan
CI: Confidence Interval
COR: Crude Odds Ratio
FANC: Focused Antenatal Care
GEE: Generalized Estimating Equation
PNC: Post-natal Care
